# Intracranial hemangioblastomas in children: Clinical-radiological characteristics, microsurgical strategy, and long-term outcomes in a 10-year single-center cohort

**DOI:** 10.1016/j.bas.2026.106085

**Published:** 2026-05-02

**Authors:** Qishuai Yu, Desheng Kong, Liang Zhang

**Affiliations:** a Department of Neurosurgery, The First Medical Center, Chinese PLA General Hospital, Beijing, China; b Department of Neurosurgery, Peking University International Hospital, Peking University, Beijing, China; c Department of Neurosurgery, National Cancer Center/National Clinical Research Center for Cancer/Cancer Hospital, Chinese Academy of Medical Sciences and Peking Union Medical College, Beijing, 100021, China

**Keywords:** Hemangiblastoma, Intracranial, Pediatric, von Hippel-Lindau, Microsurgery, Prognosis

## Abstract

**Introduction:**

Intracranial hemangioblastomas (HBs) are exceedingly rare in the pediatric population, and their clinical-radiological features, optimal treatment modalities, and long-term prognosis remain inadequately defined.

**Research question:**

We sought to characterize the clinical-radiological characteristics, treatment modalities and clarify the surgical outcomes for pediatric patients with brain HBs.

**Material and methods:**

Consecutive patients aged ≤18 years who underwent resection for intracranial HBs between 2011 and 2021 were retrospectively reviewed. Epidemiologic, clinical, neuroimaging, treatment modalities, and follow-up data were integrated and reviewed.

**Results:**

Thirty-three patients (male:female = 1.2:1; mean age 14.9 ± 2.8 years) were identified. Eighteen cases (54.5%) were sporadic, 15 (45.5%) were von Hippel-Lindau (VHL)-associated. Presenting manifestations mainly reflected posterior-fossa hypertension and cerebellar dysfunction: headache (n = 21, 63.6%), vomiting (n = 13, 39.4%), and gait instability (n = 9, 27.3%). Neuroimaging revealed a predominant cerebellar location (n = 30, 76.9%); most (n = 29, 74.4%) exhibited the classic cyst-with-enhancing-nodule architecture. VHL-associated HBs were smaller (p = 0.039) and more frequently multi-focal. Gross-total resection (GTR) achieved in 94.9% lesions. At follow-up, all recurrences (n = 4, 12.1%) occurred in the VHL cohort, yielding a 5-year progression-free survival (PFS) of 87.9% for the entire series but only 73.3% for VHL patients. Multi-focal central nervous system (CNS) and extra-CNS VHL lesions necessitated additional interventions in 53.3% of VHL cases.

**Discussion and conclusion:**

Pediatric intracranial HBs demonstrate unique features, including a higher rate of VHL association. Early surgical intervention remains the main treatment methods, ensuring favorable outcomes when performed properly. Life-long follow-up is essential, particularly for those with VHL-associated tumors, due to their higher recurrence risk.

## Highlights

• Pediatric with brain HBs has distinct clinicopathological features, have a higher relapse risk than their adult counterparts.
• Surgery can achieve satisfactory outcomes for children with brain HBs.
• Life-long follow-up is needed, especially for VHL-associated patients.

## Abbreviations

HBs: hemangioblastomas
VHL: von Hippel–Lindau
GTR: Gross-total resection
PFS: progression-free survival
CNS: central nervous system
IRB: Institutional Review Board
mRS: modified Rankin Scale
SSEP&MEP: somatosensory-evoked and motor-evoked potentials
ICG: indocyanine-green
AVM: arteriovenous malformation
EOR: extent of resection
GTR: gross total resection
STR: subtotal resection
ICP: intracranial pressure
SD: standard deviation
CSF: cerebrospinal fluid

## 1 Introduction

Intracranial hemangioblastomas (HBs) is a highly vascular, benign tumor that accounts for 1.5-2.5% of all intracranial neoplasms (Louis et al., 2021; Jankovic et al., 2024; Kuharic et al., 2018). Histologically characterized by abundant capillary networks interspersed with stromal cells, these World Health Organization (WHO) grade 1 lesions typically exhibit slow growth patterns (Louis et al., 2021; Kuharic et al., 2018). Although the vast majority arise within the posterior fossa-particularly the cerebellar hemispheres-the tumor may occur anywhere along the neuraxis, including the supratentorial compartment, brainstem, and spinal cord (Jankovic et al., 2024; Kuharic et al., 2018; Wanebo et al., 2003; Launbjerg et al., 2017). Neurological deficits results from both the solid nodule part and its associated peritumoral cyst, which together exert mass effect on eloquent posterior fossa structures, potentially causing obstructive hydrocephalus, cranial nerve palsies, or cerebellar dysfunction (Kuharic et al., 2018; Wanebo et al., 2003; Launbjerg et al., 2017).

Brain HBs manifests in two distinct biological contexts with different natural histories. Approximately 70-80% are sporadic, presenting as solitary lesions, whereas 20-30% develop in the setting of von Hippel-Lindau (VHL) disease, an autosomal-dominant cancer predisposition syndrome characterized by germline loss-of-function variants of the VHL tumor-suppressor gene on chromosome 3p25 (Jankovic et al., 2024; Kuharic et al., 2018; Wanebo et al., 2003; Yin et al., 2020; Fisher et al., 2002; Sharma et al., 2025). In VHL-associated cases, HBs are typically multifocal, may emerge *de novo* throughout life, and exhibit unpredictable growth patterns, needing lifelong radiographic surveillance and repeated surgical interventions (Wanebo et al., 2003; Yin et al., 2020). Epidemiological data indicate that HBs incidence increases with age, peaking during the fourth and fifth decades, rendering its occurrence in the pediatric population exceptional (Launbjerg et al., 2017; Yin et al., 2020; Yang et al., 2021; Knoblauch et al., 2024; Cheng et al., 2017). Population-based studies estimate the incidence of CNS HBs in children at less than 1 per 1,000,000, constituting a rare disease entity that poses unique diagnostic and therapeutic challenges (Fisher et al., 2002; Yang et al., 2021).

The clinical and neuroimaging phenotype of intracranial HBs in children remains inadequately defined, with existing management modality largely extrapolated from adult surgical series. However, emerging evidence suggests that pediatric HBs exhibits distinct biological behavior that was different from their adult counterparts (Yin et al., 2020; Yang et al., 2021; Cheng et al., 2017). Notably, children demonstrate a higher proportion of VHL-associated disease compared to adults, with recent cohort studies reporting VHL positivity in half pediatric cases (Yang et al., 2021; Knoblauch et al., 2024; Cheng et al., 2017; Kim et al., 2006; Karabagli et al., 2007; Han et al., 2023; Tomaccini et al., 1980; Vougioukas et al., 2006). This epidemiological feature carries profound clinical implications: VHL-associated pediatric patients present with multiple lesions more frequently, and exhibit shorter symptom durations compared to sporadic cases (Yang et al., 2021; Cheng et al., 2017; Karabagli et al., 2007; Han et al., 2023). Furthermore, the phenotypic spectrum of VHL disease in children may differ from adults, necessitating comprehensive genetic counseling and surveillance protocols (Wanebo et al., 2003; Fisher et al., 2002; Knoblauch et al., 2024).

Current therapeutic guidelines for pediatric intracranial HBs are notably lacking. Microsurgical resection remains the gold standard for symptomatic intracranial HBs in adults, with gross total resection (GTR) typically conferring definitive cure in sporadic cases (Jankovic et al., 2024; Yin et al., 2020). However, the optimal surgical strategy for multifocal VHL-associated lesions in children-whether aggressive resection of all radiographically evident tumors versus symptom-directed intervention-remains controversial (Kuharic et al., 2018; Wanebo et al., 2003; Yang et al., 2021; Han et al., 2023). Similarly, the role of emerging systemic therapies, including hypoxia-inducible factor (HIF) inhibitors such as belzutifan, has been inadequately explored in the pediatric population (Duan et al., 2025). These disparities underscore the imperative to establish evidence-based, pediatric-specific diagnostic and therapeutic methods, and long-term surveillance strategies.

Given the rarity of pediatric intracranial HBs and the paucity of dedicated pediatric cohort studies, we conducted a comprehensive analysis of a single-institution 10-year experience to characterize the clinical presentation and neuroimaging features specific to pediatric intracranial HBs; delineate the microsurgical strategies employed and their immediate surgical outcomes; evaluate long-term neurological and functional outcomes. We seek to inform evidence-based management strategies tailored to the distinct biological behavior of intracranial HBs in children.

## 2 Methods

### 2.1 Study design and participants eligibility

This study was reported in accordance with the Strengthening the Reporting of Observational Studies in Epidemiology (STROBE) guidelines for observational cohort studies. This was a retrospective cohort study of all eligible pediatric patients with intracranial HBs, which was approved by the Institutional Review Board (IRB) of our hospital and conducted in accordance with the 1964 Declaration of Helsinki. Consent was obtained from all patients’ guidance included in this study. All pediatric patients undergoing resection for intracranial HBs with histopathological confirmation between October 2011 and October 2021 were enrolled.

Inclusion Criteria were: 1) age ≤18 years at the time of surgery, 2) histopathologically confirmed intracranial HBs (WHO grade 1), 3) microsurgical resection performed at our institution during the study period, 4) availability of preoperative and postoperative neuroimaging (MRI with contrast). Exclusion criteria were: 1) incomplete medical records or missing operative reports, 2) follow-up < 48 months, 3) previous radiosurgery, 4) patients with concurrent malignant brain tumors, and 5) cases with unavailable histopathological confirmation.

### 2.2 Data acquisition and variable definitions

Demographics, symptom, neuroimaging, neurological status (assessed by modified Rankin Scale, mRS), and familial history were extracted. Pre- and post-operative Magnetic Resonance Imaging (MRI, 3T) sequences were reviewed by two neuroradiologists blinded to outcomes. Tumor size was defined as the largest diameter of the solid component and the extratumoral cysts. VHL disease was diagnosed by germline analysis or fulfilled clinical criteria. Clinical diagnosis was made if a patient had at least one VHL-associated HB and a family history of VHL disease, or if they had two or more VHL-associated HBs regardless of family history (Lonser et al., 2003).

### 2.3 Surgical techniques

Surgical resection was the primary treatment modality. All operations were performed by a single pediatric neurovascular team. The operation was tailored to the location of HBs: a suboccipital craniotomy was performed for expose cerebellar HBs, a posterior midline approach was used to expose brainstem tumors, and a frontal or occipital craniotomy for exposure supratentorial HBs. Intra-operative somatosensory-evoked and motor-evoked potentials (SSEP&MEP) monitoring and indocyanine-green (ICG) angiography were routinely employed. Resection of the HBs should follow arteriovenous malformation (AVM) principles: sequential coagulation of arterial feeders, circumferential microdissection along the tumor-pial plane, *en bloc* excision of the HBs, and coagulation the draining vein at last to minimize hemorrhage.

### 2.4 Outcomes measurement

Extent of resection (EOR) was defined as GTR (no residual on 24h MRI) or subtotal resection (STR). Progression-free survival (PFS) was defined as interval from surgery to radiological recurrence or last follow-up (October 2025). Overall survival (OS) was defined as time between surgery and dead. Neurological outcome was categorized as improved, stable, or deteriorated relative to pre-operative mRS.

### 2.5 Follow-up protocol

Post-operative surveillance was conducted by clinic visits or telephone calls. Outpatient evaluations-including detailed neurological examination and mRS scoring-were scheduled at 3, 6, and 12 months after discharge and annually thereafter. Brain MRI with gadolinium was obtained at each visit in the first year; additional imaging was prompted by new or worsening symptoms. This follow-up protocol enabled real-time assessment of functional status and timely identification of local relapse or remote *de novo* lesion. The last follow-up data were collected in October 2025, yielding a minimum potential follow-up of 48 months, thereby permitting assessment of long-term surgical outcomes. All patients in the study period was followed and incorporated in this study.

## 3 Statistical analysis

Baseline demographics and clinical variables were summarized with standard descriptive statistics. Continuous variables were reported as mean ± standard deviation (SD), and categorical variables were expressed as percentages and frequencies. Categorical parameters were performed using the χ^2^ test or Fisher's exact test, and continuous variables were compared with the two-sample Students' *t*-test or Wilcoxon rank-sum test. Kaplan-Meier analysis was used to estimate PFS. A two-tailed p < 0.05 was considered significant. Analyses were conducted with SPSS software (version 28.0, IBM Corp., Armonk, NY, USA).

## 4 Results

### 4.1 Baseline characteristics

**Fig. 1:**
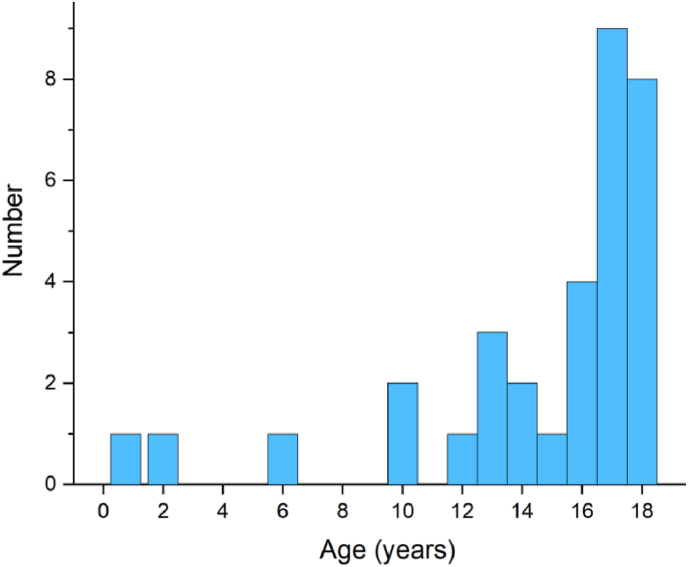
Age distribution of 33 children with brain HBs.

This cohort consisted of 33 pediatric patients (male-to-female ratio 1.2:1) with a mean age at diagnosis of 14.9 ± 4.6 years (range: 9 months-18 years) (Fig. 1). Eighteen patients (54.5%) exhibited sporadic HBs, whereas 15 (45.5%) harbored VHL-associated disease.

The most common presenting symptoms were headache (n = 21, 63.6%), vomiting (n = 13, 39.4%), and gait ataxia (n = 9, 27.3%). Additional manifestations included vertigo (n = 7, 21.2%), motor problems (n = 6, 18.2%), visual impairment (n = 2, 6.1%), and epileptic seizures (n = 1, 3.0%). These symptoms are typically related to increased intracranial pressure (ICP) and cerebellar dysfunction, which are common manifestations of posterior fossa tumors. The mean duration of symptoms was 2.8 ± 5.8 months (range: 0.1-18 months). Preoperative neurological status, quantified by the mRS, was predominantly grade 2 or 3 (Table 1).

**Table 1:** Comparison of characteristics of sporadic and VHL-associated brain HBs.

Variable	Total, N (%)	Sporadic, N (%)	VHL-associated, N (%)	P value
*Clinical characteristics*				
Case number	33	18	15	
Age (yrs)*	14.9 ± 4.6 (0.75-18)	15.2 ± 4.4 (0.75-18)	14.6 ± 5.0 (2-18)	0.714
Sex				0.407
Male	18 (54.5)	11 (61.1)	7 (46.7)	
Female	15 (45.4)	7 (38.9)	8 (53.3)	
Symptoms				
Headache	21 (63.6)	12 (66.7)	9 (60.0)	0.692
Nausea or vomiting	13 (39.4)	7 (38.9)	6 (40.0)	0.948
Ataxia	9 (27.3)	7 (38.9)	2 (13.3)	0.134
Vertigo	7 (21.2)	4 (22.2)	3 (20.0)	1.000
Motor deficit	6 (18.2)	5 (27.8)	1 (6.7)	0.186
Visual problem	2 (6.1)	2 (11.1)	0 (0)	0.489
Seizure	1 (3.0)	1 (5.6)	0 (0)	1.000
Hydrocephalus	15 (45.5)	9 (50.0)	6 (40.0)	0.566
Duration of symptoms (mos)^a^	2.8 ± 5.8 (0.1-18)	2.8 ± 5.7 (0.3-9)	2.7 ± 6.2 (0.1-18)	0.976
Pre-op mRS				0.948
1	3 (9.1)	2 (11.1)	1 (6.7)	
2	11 (33.3)	5 (27.8)	6 (40.0)	
3	16 (48.5)	9 (50.0)	7 (46.7)	
4	3 (9.1)	2 (11.1)	1 (6.6)	
Synchronous spinal HBs	9 (27.3)	0 (0)	9 (60.0)	**<0.001**
*MRI features*				
Multi-lesions	3 (9.1)	1 (11.1)	2 (13.3)	0.579
Location				0.289
Cerebellum	30 (76.9)	13 (68.4)	17 (85.0)	
Brainstem	6 (15.4)	3 (15.8)	3 (15.0)	
Supratentorial (frontal/occipital lobe)	3 (7.7)	3 (15.8)	0 (0)	
Maximum size (cm)*	2.8 ± 1.8 (0.5-6)	3.2 ± 1.8 (1.5-6)	1.0 ± 0.7 (0.5-1.5)	**0.039**
Maximum volume (cm^3^)*	14.3 ± 24.2 (0.06-60)	17.8 ± 26.1 (1.4-60)	1.2 ± 0.2 (0.06-2.3)	**0.047**
Presence of cyst	25 (64.1)	12 (63.2)	13 (65.0)	0.905
Hemorrhage	4 (10.3)	4 (21.1)	0 (0)	**0.047**
Resection				1.000
GTR	37 (94.9)	18 (94.7)	19 (95.0)	
STR	2 (5.1)	1 (5.3)	1 (5.0)	

Abbreviations: *yrs*, years; *mos*, months; *mRS*, modified Rankin scale; *HBs*, hemangioblastomas; *VHL*, Von Hippel-Lindau; *GTR*, gross total resection; *STR*, subtotal resection.

a They were expressed in mean ± standard deviation.

### 4.2 Imaging examinations

MRI were conducted for all children, three patients (9.1%) had multiple HBs. Thirty tumors (76.9 %) were located within the cerebellar parenchyma, six (15.4 %) in the brainstem, and three (7.7 %) in supratentorial compartments. The classic cystic-solid morphology-defined as an enhancing nodule surrounded by a large non-enhancing cyst-was present in 34 lesions (87.2 %). Among VHL-associated cases, nine (60.0%) exhibited synchronous spinal HBs, and four (26.7%) demonstrated peritumoral hemorrhage. Mean maximal diameter was 2.8 ± 1.8 cm (range: 0.5-6.0 cm) and tumor volume 14.3 ± 24.2 cm^3^ (range: 0.06-60 cm^3^), which were greater in sporadic compared with VHL-associated HBs (diameter 3.2 ± 1.8 cm vs 1.0 ± 0.7 cm, p = 0.039; volume 17.8 ± 26.1 cm^3^ vs 1.2 ± 0.2 cm^3^, p = 0.047). No inter-group differences emerged in age, sex, symptom duration, baseline mRS, or lesion location (Table 1).

## 5 Surgical details and intraoperative findings

GTR was achieved in 37 of 39 lesions (94.9%), while STR was performed in 2 (5.1%) confirmed by postoperative MRI. One patient with frontal lesion which was close to fiber tract, and GTR was deemed unsafe. One lesion was adhered tightly to the brainstem without an obvious gliotic plane, and GTR was unachievable. The mean blood loss was 356.7 ± 130 (range: 50-1900) ml, four children (12.1%) received autologous blood transfusion, and 1 child (3.0%) experienced homologous blood transfusion.

## 6 Surgical outcomes

Two children (6.1%) suffered a fever assumed to be meningitis, and they were treated with lumbar puncture and antibiotics, and all were recovered before discharge. No patients experienced cerebrospinal fluid (CSF) leakage or wound infection. One patient (3.0%) with cerebellar and brainstem HBs suffered from hematoma and received conservative treatment and recovered before discharge.

Twenty patients (60.6%) experienced improvements in neurological function, while eleven patients (33.3%) remained unchanged. Two (6.1%) with cerebellar and brainstem HBs experienced neurological deterioration immediately after surgery, manifested as coma, and they received frontal horn puncture, one recovered and was in good condition and one was dead (Table 2).

**Table 2:** Functional changes of patients with brain HBs.

mRS	Pre-op	Post-op	Follow-up
0	0 (0)	2 (6.1)	22 (66.7)
1	3 (9.1)	8 (24.2)	5 (15.1)
2	11 (33.3)	16 (48.5)	3 (9.1)
3	16 (48.5)	6 (18.2)	2 (6.1)
4	3 (9.1)	0 (0)	0 (0)
5	0 (0)	1 (3.0)	0 (0)
6	0 (0)	0 (0)	1 (3.0)

Abbreviations: *mRS*, modified Rankin scale; *HBs*, hemangioblastomas; *pre-op*, pre-operative; *post-op*, post-operative.

## 7 Long-term prognosis

During a mean follow-up period of 127.1 ± 40.7 (range: 48-168) months, recurrence at the site of the original tumor was detected in 4 children (12.1%). They were in the VHL group, and opted a second surgery after an average of 41.3 (5-86) months after the first surgery. Children in the VHL group underwent more repeated surgeries for spinal HBs (n = 8, 53.3%), or other VHL-associated tumors (n = 5, 33.3%) beyond the central nervous system (CNS).

**Fig. 2:**
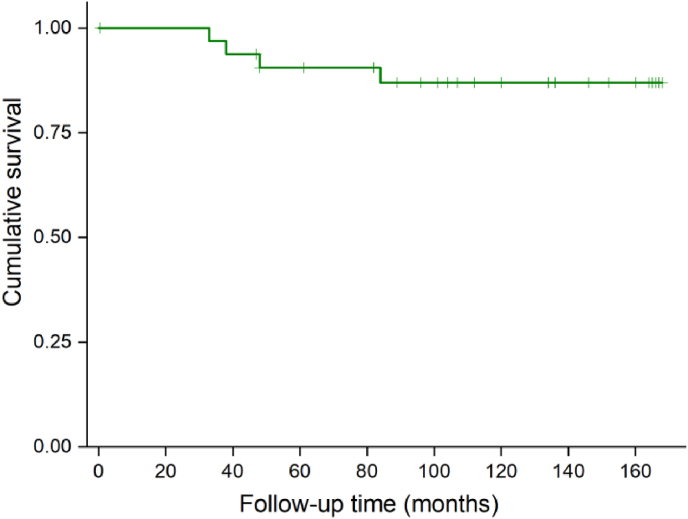
Kaplan-Meier curve for recurrence-free survival of brain HBs in pediatric patients.

The 5-year PFS rate for the entire cohort was 87.9% (Fig. 2). This indicates a favorable prognosis for pediatric patients with intracranial HBs, particularly when GTR is achieved. Disability calculated by the mRS was grade 0 in 22 (66.7%), grade 1 in 5 (15.1%), grade 2 in 3 (9.1%), grade 3 in 2 (6.1%) patients, and grade 6 in 1 (3.0%) patient at the last follow-up (Table 2).

## 8 Discussion

This is one of the largest reported cohorts of children with brain HBs. This study systematically delineates the clinical presentation, management strategies, and treatment outcomes of pediatric patients with intracranial HBs. Our findings indicate that pediatric patients, particularly those with VHL disease, characteristically manifest smaller tumor size and volume, increased multifocal tumors, and a higher propensity for repeated surgical interventions compared to their sporadic counterparts. Surgical resection remains the cornerstone of treatment, with GTR serving as favorable clinical outcomes.

## 9 Demographics

Intracranial HBs are infrequently encountered in the pediatric population, and their true incidence remains unclear. In our series, the mean age at presentation was 14.9 years, with the majority of patients aged 10-18 years, suggesting that the incidence of HBs increases with advancing age and rarely manifests before the first decade of life (Yang et al., 2021; Cheng et al., 2017; Han et al., 2023). The sex distribution of pediatric brain HBs remains a subject of debate. Notably, we observed a slight male predominance (54.5%), consistent with the gender distribution reported in adult populations (Jankovic et al., 2024; Kuharic et al., 2018; Yin et al., 2020). In this cohort, approximately half of the children (45.5%) harbored VHL-associated disease, which was in accordance with previous findings in pediatric patients (Yang et al., 2021; Cheng et al., 2017; Karabagli et al., 2007; Han et al., 2023) but higher than that observed in adult cohorts (Jankovic et al., 2024; Kuharic et al., 2018; Yin et al., 2020). However, referral bias may exist, as VHL-associated cases may be overrepresented, leading to an overestimation of VHL prevalence in our series. A greater proportion of sporadic patients was observed in our series, suggesting that the tumorigenesis of HBs in children may differ from that in adults and may involve molecular processes unrelated to the VHL tumor suppressor pathway (Fisher et al., 2002).

Despite their histologically benign nature, pediatric HBs can may cause significant morbidity via mass effect. In the present cohort, the predominant clinical manifestations included headache, vomiting, and gait ataxia-symptoms attributable to the predilection of these tumors for the cerebellum, their propensity for rapid volumetric expansion, and their tendency to obstruct CSF circulation. MRI demonstrated superior capability in delineating cystic architectural patterns and contrast enhancement characteristics, underscoring its indispensable role in diagnostic evaluation. In this study, three supratentorial HBs were encountered, which are extremely rare and were observed exclusively in sporadic patients. This may pose potential diagnostic challenges and necessitate careful differential diagnosis to prevent misdiagnosis as previous reports (Kim et al., 2006; Tomaccini et al., 1980).

The anatomical distribution of HBs in pediatric patients is similar as that observed in adult cohorts (Jankovic et al., 2024; Kuharic et al., 2018; Yin et al., 2020; Mills et al., 2012). However, we observed that pediatric HBs exhibited larger size and volume compared to adult lesions (Jankovic et al., 2023), suggesting accelerated growth patterns in the pediatric population. The pathogenesis of pediatric intracranial HBs remains incompletely understood, likely reflecting complex interactions between genetic factors and abnormal angiogenesis. In this study, we found that VHL-associated HBs were smaller compared to sporadic cases. We propose two mechanisms to account for this size difference. First, surveillance bias exists, as VHL patients undergo lifelong MRI screening, leading to earlier detection at smaller sizes before substantial solid growth. Second, biological divergence may contribute, as VHL germline mutations drive early cystogenesis via HIF-1α/vascular endothelial growth factor (VEGF)-mediated vascular permeability and exudative fluid accumulation, potentially decoupling cyst growth from solid nodule proliferation. VHL-associated stromal cells exhibit distinct angiogenic profiles favoring cyst formation over cellular expansion.

Factors that may trigger symptoms in smaller VHL-associated tumors, including location, cyst volume, and the potential role of the VHL pathway. Even smaller tumors in critical anatomical locations can cause symptoms due to mass effect or obstruction of CSF pathways. The presence and volume of associated cysts, rather than the solid tumor component alone, can contribute significantly to mass effect and symptom onset. Because pediatric posterior fossa HBs often present with disproportionately large cysts relative to the mural nodule, even small solid components can produce significant mass effect in the constrained posterior fossa. The VHL gene itself plays a role in angiogenesis and cellular proliferation. Alterations in this pathway might influence tumor growth dynamics and presentation, even leading to smaller tumors that are nonetheless clinically significant due to location and cyst formation.

## 10 Management strategies and outcomes

The optimal treatment method of pediatric HBs was mainly extrapolation from their adult counterpart due to the lack of relevant studies (Han et al., 2023; Jankovic et al., 2023; Harbi and Aschner, 2025). The majority of patients in our cohort demonstrated substantial symptomatic improvement and favorable postoperative prognoses following microsurgical resection, supporting the primacy of surgical intervention in this population. Superficially located tumors with well-demarcated boundaries allow straightforward total resection and are associated with low recurrence rates. Conversely, deeply located lesions closely related to eloquent structures-such as brainstem nuclei and critical white matter tracts-present great surgical challenges and carry elevated risks of postoperative neurological morbidity. As demonstrated in this study, both patients who experienced postoperative neurological deterioration harbored cerebellar and brainstem HBs.

We advocate for early surgical intervention before significant neurological deficits occurred, particularly in patients with sporadic disease. All children except one in sporadic group experienced stable or improved functional status (mRS scores 0-1) following surgery. Timely resection can effectively stop tumor progression and mitigate irreversible neural injury. However, patients with VHL-associated HBs frequently present with concurrent spinal lesions and multifocal intracranial HBs, necessitating multiple surgical procedures and potentially accumulating neurological deficits. The optimal surgical timing in patients with multiple VHL-associated tumors remains debatable (Han et al., 2023). A symptom-directed approach appears reasonable, wherein lesions responsible for current symptom are resected firstly, while asymptomatic lesions may be managed expectantly until radiological progression or new symptoms emerge. In our VHL cohort, we prioritized resection of symptomatic lesions, deferring intervention for asymptomatic tumors until evidence of radiological progression or clinical deterioration.

In general, the neurological outcomes for pediatric with brain HBs were generally favorable, with 93.9% of patients demonstrating stable or improved functional status. The surgical outcomes were comparable to those reported in previous studies (Cheng et al., 2017). Nevertheless, the high recurrence rate observed in the VHL-associated group represents a significant clinical challenge. Our center is a high-volume referral institution where complex VHL-associated or recurrent cases are frequently admitted; we acknowledge that this may have led to an overestimation of recurrence rates in our cohort. Lesion relapse and repeated surgical interventions may cumulatively exacerbate neurological deficits. However, our data demonstrate that a repeated surgery can provide benefit for patients experiencing recurrence. As surgical technologies (intraoperative navigation, fluorescence guidance) evolved over the study period, this may have influenced surgical outcomes and contributed to improved results.

Pediatric patients with brain HBs require long-term surveillance, even in sporadic cases, given their extended life expectancy. Future multi-center prospective investigations with larger sample sizes are imperative to refine treatment strategies and optimize management of this rare pediatric neoplasm.

## 11 Limitations

This study has several limitations. First, its retrospective, single-centre design introduce selection and information biases. Complex VHL-associated or recurrent cases may be overrepresented compared with community settings, potentially leading to an overestimation of VHL prevalence and recurrence rates in our cohort. Second, because pediatric cerebral HBs are exceptionally rare, only a small case series could be assembled over the study interval, yielding low statistical power and limiting the robustness of subgroup analyses. The small sample size may introduce bias in the interpretation of results, leading to a potentially prejudiced conclusion. Third, the 10-year study period spanned evolving surgical technologies, which may have affected comparisons of surgical outcomes across time points. Lastly, the natural history of the disease in childhood remains poorly characterized; prospective, multi-centre registries with lifelong follow-up are required to quantify lifetime tumor risk. Despite these limitations, this represents, to our knowledge, the largest reported cohort of children with brain HBs.

## 12 Conclusion

Pediatric brain HBs represents a rare vascular neoplasm, with distinct clinicopathological features differentiating it from adult presentations. Pediatric patients with brain HBs appear to have a higher relapse risk than their adult counterparts, thus indicating a necessity for life-long follow-up, especially for VHL-associated patients. Surgery can achieve satisfactory outcomes, and should be considered before irreversible neurological deterioration occurred.

## Consent to participate

Informed consent was obtained from all individual participants included in the study.

## Ethical approval and consent to participate

All procedures performed in studies involving human participants were in accordance with the ethical standards of the institutional and/or national research committee by the 1964 Helsinki Declaration and its later amendments or comparable ethical standards.

## Availability of data and material

Not applicable.

## Author's contributions

Qishuai Yu, Desheng Kong, and Liang Zhang collected the data and wrote the draft of the manuscript text and prepare the figures and tables. Liang Zhang revised the manuscript. All authors reviewed the manuscript.

## Funding

No financial interests or potential conflict of interest is involved in this research.

This study was supported by CAMS Innovation Fund for Medical Sciences (CIFMS) (2024-I2M-C&T-B-055).

## Declaration of competing interest

The authors declare that they have no known competing financial interests or personal relationships that could have appeared to influence the work reported in this paper.
